# Enhanced Therapeutic Potential of Irreversible Electroporation under Combination with Gold-Doped Mesoporous Silica Nanoparticles against EMT-6 Breast Cancer Cells

**DOI:** 10.3390/bios13010041

**Published:** 2022-12-27

**Authors:** Yixin Jiang, Ratchapol Jenjob, Su-Geun Yang

**Affiliations:** Department of Biomedical Science, BK21 FOUR Program in Biomedical Science and Engineering, College of Medicine, Inha University, Incheon 22212, Republic of Korea

**Keywords:** gold nanoparticle, mesoporous silica nanoparticles, irreversible electroporation, lipid peroxidation, reactive oxygen species, cell permeability, breast cancer

## Abstract

Irreversible electroporation (IRE) is a non-thermal tumor ablation technique that delivers short pulses of strong electric fields to cancer tissues and induces cell death through the destruction of cell membranes. Here, we synthesized gold-doped mesoporous silica nanoparticles (Au-MSNs) via incipient wetness impregnation and evaluated the therapeutic potentials of combination therapy with IRE. The fabricated Au-MSNs had around 80–100 nm of particle size and were successfully end-doped with Au nanoparticles. Combination treatment of IRE (800 V/cm) and Au-MSNs (100 μg/mL) increased cell membrane permeability by 25-fold compared with single IRE treatment. Cellular reactive oxygen species (ROS) and lipid peroxidation of EMT-6 cells were significantly increased by 14- and 265-fold, respectively, under combination treatment of IRE (800 V/cm) and Au-MSNs (100 µg/mL). Cytotoxic cell death increased by 28% under a combination treatment of IRE (800 V/cm) and Au-MSNs (100 ug/mL) over single IRE. Our studies suggest that the combination treatment of IRE with Au-MSNs can enhance the therapeutic efficacy of IRE for breast cancer.

## 1. Introduction

Irreversible electroporation (IRE) is an ablation technique that is currently being developed for cancer adjuvant therapy [1,2]. IRE transmits microsecond electrical pulses across the target tissue, creating permanent nanoscale pores on the cell membrane. In this way, IRE disrupts cell membrane integrity and eventually causes a complete collapse of cellular homeostasis and cell death. The amplitude and duration of electric stimuli should be closely controlled to get the most effective ablation of tumor tissue. IRE is now being studied for the treatment of prostate cancer, liver cancer, pancreatic cancer, and kidney cancer [3]. However, the reported clinical results of IRE for cancer therapy remain contentious, and scientists are currently working to incorporate more robust treatment modalities into IRE.

The most prominent cellular response after IRE treatment is the explosive generation of reactive oxygen species (ROS), and this response appears to be the primary cause of the total collapse of cellular homeostasis. ROS, specifically peroxides, superoxide, hydroxyl radical, and singlet oxygen, cause oxidative damage to proteins, DNA, fatty acids, and other biological molecules [4,5,6]. ROS is related to various types of cell death, including apoptosis, necrosis, and ferroptosis. ROS is largely implicated in chemotherapy, photodynamic therapy, sonodynamic therapy, and IRE for cancer treatments [5,7,8,9]. Therefore, combined treatment of IRE with other therapeutic modalities may display a synergistic effect for cancer therapy.

Oxygen evolution reactions (OERs) are the process of generating oxygen molecules (O_2_), generally from water, through a chemical reaction. OER is a critical step in energy storage and conversion, particularly in water electrolysis. To minimize energy consumption and improve energy conversion efficiency, rare metals and their oxide compounds (Ir, Ru, IrO_2_, and RuO_2_) are being used extensively in the OER process. Gold (Au), in the form of gold nanoparticles (AuNPs) as electrocatalysts, serves as an efficient heterogeneous catalyst for OER. AuNPs under the pulsed electrical field (EF) transfer the electron energy to water, activate the electron energy state of water molecules, and precipitate the chemical reactions. Interestingly, scientists proved that water molecules are the main player in pore formation on the cell membrane under an IRE treatment [10]. Thus, combined IRE treatment with AuNPs may enhance the therapeutic efficacy of IRE on cancers.

Additionally, AuNPs work as virtual microelectrodes with high electro-conductivity (~ 4.5 × 10^6^ S/m), converge electric pulses, and increase the perforation of the cell membrane [11]. AuNPs have been studied for adjuvant modalities in various therapeutic targets and have shown interesting synergistic efficacy [12,13,14,15]. Yingbo Zu and his research group estimated the transfection efficiency of plasmid DNA using highly conductive AuNPs [16]. They demonstrated that AuNPs enhanced electroporation performance in mammalian cells and found that the transfection efficiency was affected by the size, concentration, and mixing ratio of AuNPs. Pratx and his research group proved combination design of radiation therapy and AuNPs displayed a strong synergistic effect in terms of radio sensitization [17]. Wu and his research group reported that AuNPs disrupted cellular ROS homeostasis after trafficking into the mitochondria and induced apoptotic cell death [18].

Cellular oxidative stress, including ROS and lipid peroxidation, is observed at approximately 200–1000 V/cm electric field application. ROS production is dependent on electric power density and frequency of electric pulses for IRE application [2]. Lipid peroxidation of polyunsaturated fatty acids occurs during the burst release of ROS and subsequent cellular bioreactions (e.g., formation of hydroperoxide lipids on membranes). ROS and lipid peroxidation play a critical role in cellular metabolism pathways and can act as signals for inflammation, proliferation, and program cell death.

**Scheme 1:**
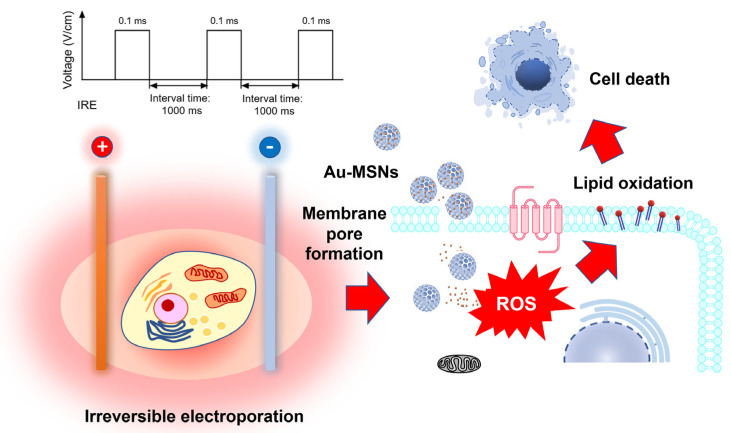
Gold-doped mesoporous silica nanoparticles (Au-MSNs) enhance the therapeutic potential of irreversible electroporation (IRE). Combination treatment of IRE and Au-MSNs more abundantly produced cellular reactive oxygen species (ROS) than single IRE treatment, effectively induced the oxidation of phospholipid of cell membrane and caused the cancer cell death.

We hypothesized that AuNPs enhance the therapeutic potential of IRE and eventually increase the antitumor efficacy of IRE (Scheme 1). To prove this hypothesis, we developed the Au-doped mesoporous silica nanoparticles (Au-MSNs) to enhance the efficiency of IRE and investigated its potential as a new therapeutic strategy for breast cancer. We evaluated the in vitro cytotoxicity of Au-MSNs in combination with IRE on EMT-6 breast cancer cells. The cellular ROS generation and lipid peroxidation were measured using DCFH-DA and BODIPY 581/591 C11 dyes, respectively, under IRE treatment with different concentrations of Au-MSNs. We also assessed the cell membrane permeability of EMT-6 cancer cells in the presence of FITC-labeled Au-MSNs under IRE treatment, along with therapeutic cytotoxicity.

## 2. Materials and Methods

### 2.1. Materials and Reagents

Pluronic F127 (PF127, CAS No.: 9003-11-6), hexadecyltrimethylammonium chloride (CTAC, CAS No.: 112-027), and tetraethyl orthosilicate (TEOS, CAS No.: 78-10-4) were obtained from TCI (Tokyo, Japan). Sodium hydroxide (1 M) was obtained from Duksan Co. (Gyeonggi-do, Korea). Gold (III) chloride trihydrate (HAuCl_4_·3H_2_O, CAS No.: 16961-25-4) and 2ʹ,7ʹ-dichlorofluorescin diacetate (DCF-DA, CAS No.: 4091-99-0) were purchased from Sigma-Aldrich (St. Louis, MO). BODIPY 581/591 C11 (CAS No.: D3861) was received from Thermo Fisher Scientific (Waltham, MA, USA).

### 2.2. Synthesis of Mesoporous Silica Nanoparticles

Mesoporous silica nanoparticles (MSNs) were synthesized using PF127 and CTAC as a cosurfactant [19,20]. A mixture of CTAC (200 mg) and PF127 (50 mg) was dissolved in distilled water (96 mL), and pH was adjusted using 1 M NaOH (1.4 mL) with vigorous stirring at 80 °C for 1 h. Then, 0.75 mL of TEOS was added to the solution and stirred further at 80 °C overnight. Samples were then collected through centrifugation (10,000 rpm, 30 min). The surfactant in the MSNs was removed by centrifugal purification after mixing with 1 *v*/*v*% HCl/MeOH solution. The purified samples were redispersed with distilled water and dried at 60 °C.

### 2.3. Synthesis of Au-MSNs 

Au-MSNs were synthesized through incipient wetness impregnation [21]. Briefly, a gold (III) chloride trihydrate (HAuCl_4_·3H_2_O) solution (50 and 120 µg/mL) was added to 50 mg of MSNs and calcinated at 500 °C for 4 h. The applied amount of HAuCl_4_ of 50 and 120 µg/mL was denoted as 5 and 12%, respectively. The obtained Au-MSNs were centrifuged, washed with distilled water thrice (10,000 rpm, 30 min), and dried under vacuum at 50 °C for 6 h [22]. Field emission transmission electron microscopy (FE-TEM, FEI Co., Hillsboro, OR, USA) was used for the morphological characterization of Au-MSNs. Energy dispersive x-ray (EDX) spectroscopy analysis was conducted to determine the elementary distribution of Si, O, and Au. The size distribution of Au-MSNs was measured using a Zetasizer Nano ZS system (Malvern Instruments, Southborough, MA, USA). The ultraviolet–visible (UV-Vis) absorption spectra of MSNs and Au-MSNs were obtained with a SpectraMax M5 microplate reader (Molecular Devices, San Jose, CA, USA).

### 2.4. Irreversible Electroporation Treatment of EMT-6 Cells

EMT-6 cells (a mouse breast cancer cell line, American Type Culture Collection, Manassas, VA) were used for our in vitro study. The cells were cultured in Dulbecco’s modified Eagle’s medium (DMEM) containing 10% fetal bovine serum, 1% penicillin, and 1% streptomycin at 37 °C and 5% CO_2_. The cells with 70~80% of confluency were introduced to IRE treatment. IRE was performed using a BTX 830 electroporator (ECM830 Square Wave Electroporation System; BTX, Syngen Biotech, Wroclaw, Poland). Adherent cell electrodes with an electrode gap of 5 mm (Syngen Biotech, Wroclaw, Poland) were used for in vitro analysis. Experiments were conducted under standard IRE protocols: 20 electric pulses at 500 or 800 V/cm for 0.1 ms and 1 Hz at a pulse interval of 1 ms.

### 2.5. Evaluation of Cell Membrane Permeability

EMT-6 cells were seeded into the 6-well plates at 2 × 10^5^ cells per well and incubated at 37 °C for 12 h. EMT-6 cells were pretreated with Au-MSNs (0, 50, and 100 µg/mL). After 2 h of incubation, EMT-6 cells were further treated with IRE (500 or 800 V/cm, 20 pulses, 0.1 ms, 1 Hz, and pulse interval: 1 ms) and washed with 1 mL of Dulbecco’s phosphate-buffered saline (DPBS) three times. Then, the cells were incubated in 0.75 μg/mL of fluorescein isothiocyanate-dextran (FITC-dextran, m.w.; 10 kDa) for 30 min at 37 °C, washed with 1 mL of DPBS and fixed with 4% paraformaldehyde for microscopic observation.

### 2.6. Cellular Uptake of FITC-Au-MSNs

Cellular uptake of Au-MSNs under IRE treatment was assessed on RMT-6 cells. FITC-labeled Au-MSNs (FITC-Au-MSNs) were prepared in the dark. EMT-6 cells were seeded in six-well culture plates (2 × 10^5^ cells per well) and incubated overnight. Then cells were co-treated with FITC-Au-MSNs (50 µg/mL) and IRE. After 1 h of incubation, the uptake of FITC-Au-MSNs was observed using a fluorescence microscope and analyzed with ImageJ (Java 1.8.0_112, 64-bit).

### 2.7. Cellular ROS Staining

ROS content in EMT-6 cells was evaluated using DCF-DA, a cell-permeable fluorogenic dye. EMT-6 cells were seeded into six-well plates at 2 × 10^5^ cells per well at 37 °C for 12 h and detached via trypsinization (0.025% trypsin and 0.02% EDTA). Cells were incubated in serum-free DMEM containing Au-MSNs (0, 50, and 100 µg/mL) at 37 °C for 4 h, subjected to a PEF (20 pulses of 500 or 800 V/cm for 0.1 ms and 1 Hz at a pulse interval of 1 ms), and washed with 1 mL of DPBS three times to determine cellular ROS production. The cells were incubated in a working solution of 10 μM DCF-DA in DPBS in the dark for 30 min and observed under a fluorescent microscope.

### 2.8. Evaluation of IRE-Induced Lipid Peroxidation

BODIPY 581/591 C11 (lipid peroxidation sensor dye, Invitrogen™, ThermoFisher Scientific, Waltham, MA, USA) was used to evaluate lipid peroxidation in EMT-6 cells. EMT-6 cells were seeded in six-well plates at a concentration of 2 × 10^5^ cells per well, incubated at 37 °C for 12 h. Then, cells were incubated with different concentrations of Au-MSNs (0, 50, and 100 μg/mL) for 2 h and subjected to IRE treatment (20 pulses of 500 or 800 V/cm for 0.1 ms and 1 Hz at a pulse interval of 1 ms). Right after the IRE treatment, EMT-6 cells were incubated with 5 µM BODIPY 581/591 C11 at 37 °C for 1 h. Cellular lipid peroxidation was observed on a fluorescence microscope after washing 3 times with phosphate-buffered saline (PBS) buffer and analyzed with ImageJ.

### 2.9. Live/Dead Cell Staining

Live/dead cell staining was conducted using calcein acetoxymethyl ester (Calcein AM, green) and ethidium homodimer-1 (EthD-1, red), as described in previous studies [23]. For the study, EMT-6 cells were seeded into six-well plates at 2 × 10^5^ cells per well and maintained at 37 °C for 12 h. Cells were treated with a combination protocol of Au-MSNs (0, 50, and 100 µg/mL) and IRE (20 pulses at 500 or 800 V/cm for 0.1 ms and 1 Hz at a pulse interval of 1 ms) and washed with 1 mL of DPBS three times. The cells were incubated in a mixture of 2 μM Calcein AM and 4 μM EthD-1 in the dark for 15 min. Cells were observed under a fluorescence microscope with relevant controls to confirm the therapeutic cytotoxicity of the combined treatment of IRE with Au-MSNs. Additionally, the cytotoxicity of Au-MSNs against EMT-6 cells was observed using a CCK-8 kit.

### 2.10. Statistical Analysis

Data were represented as mean ± SD (n = 3). Statistical significance was described as * p < 0.1, ** p < 0.05, and *** p < 0.01 (Two-way analysis of variance (ANOVA) and Student’s *t*-test).

## 3. Results and Discussion

### 3.1. Characteristics of Au-MSNs

**Figure 1:**
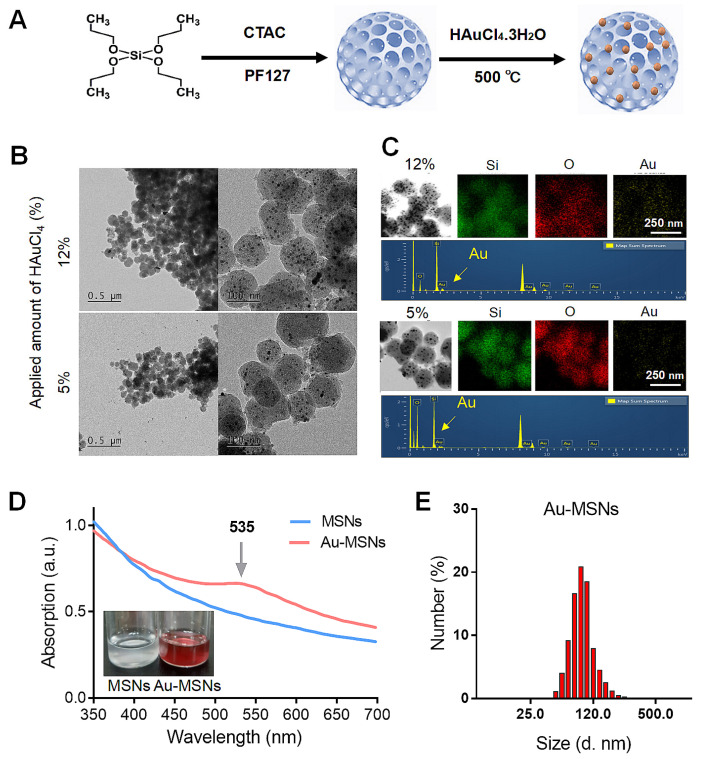
Characterizations of Au-MSNs. (**A**) Schematic illustration for synthesis of Au-MSNs. (**B**) Transmission electron microscopy (TEM) images of Au-MSNs (scale bar = 100 and 500 nm). (**C**) TEM and energy-dispersive X-ray spectroscopy (TEM EDX) mapping images of Au-MSNs with 5 wt% (50 µg/mL) or 12 wt% (120 µg/mL) of HAuCl_4_.3H_2_O for Au, Si, and O, respectively. (**D**) Ultraviolet–visible (UV-vis) absorption spectra of MSNs and Au-MSNs. (**E**) Dynamic light scattering (DLS) measurement of Au-MSNs.

Au-MSNs were prepared by incipient wetness impregnation at high temperatures (Figure 1A). As shown in Figure 1B, Au-MSNs maintained the mesoporous structure after the Au-doping. AuNPs were randomly distributed on the MSNs. The particle size of Au-MSNs, observed by the TEM, was around 80 to 100 nm. The mean diameters of MSNs and Au-MSNs measured by dynamic light scattering analysis were 92.29 ± 18.76 and 98.37± 20.53 nm, respectively (Figure 1E and Figure S1). TEM images revealed that the higher amount of HAuCl_4_ applied, the higher Au NPs obtained on the MSN template. EDX technique agreed with the TEM images in which 5% Au-MSNs and 12% Au-MSNs contained 3.31% and 11.48% by weight percentage of AuNPs, respectively (Figure 1C and Tables S1 and S2). The doping amount of AuNPs on the MSNs was relevant to the applied amount of Au elements (5% Au-MSNs and 12% Au-MSNs).

Spectroscopic observation proved the typical characteristics of the Au-MSNs. First, the successful doping of AuNPs on the MSNs was assumed by the color change of the solution from transparent to light pink (Figure 1D inset). UV-Vis spectra showing a typical plasmonic peak range from 500 to 580 nm with a maximum absorbance peak at 535 nm strongly suggested that AuNPs were successfully doped into MSNs (Figure 1D).

### 3.2. Enhanced Cellular Membrane Permeability under Combination Treatment

**Figure 2:**
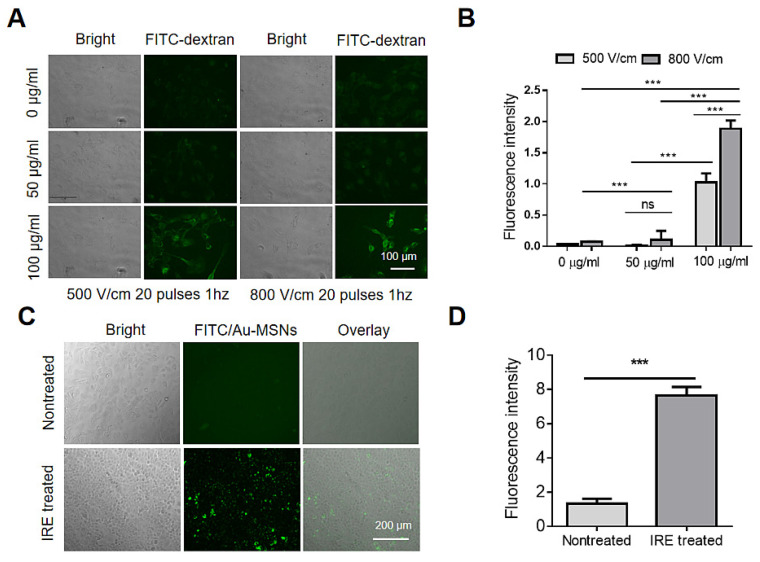
The enhanced cellular permeability of EMT-6 cells under IRE treatment with Au-MSNs. (**A**) Fluorescence microscopic images of EMT-6 cells show that IRE-treatment (500 V/cm and 800 V/cm) with Au-MSNs (50 μg/mL and 100 μg/mL) increased cell membrane permeability of EMT-6 cells. Membrane permeability was observed using fluorescein isothiocyanate-dextran (FITC-dextran). (**B**) Analysis of fluorescence intensity. Co-treatment of EMT-6 cells with IRE (800 V/cm) and Au-MSNs (100 μg/mL) increased the cellular permeation of FITC-dextran by 25-fold. (**C**) The enhanced cellular uptake of FITC/Au-MSNs under IRE treatment (800 V/cm). Fluorescence microscopic images suggest IRE treatment of EMT-6 cells dramatically increased the uptake of FITC/Au-MSNs. (**D**) Analysis of fluorescence intensity of EMT-6 cells. Data proved IRE treatment of EMT-6 cells increased the uptake of FITC/Au-MSNs by 7-fold. (Data are provided as means ± standard deviation. Data were tested for significance using Student’s *t*-test and a two-way ANOVA. Significant differences are indicated by *** p < 0.01. *n* = 3–5).

In this study, we observed cellular membrane permeability using FITC-dextran under the combined treatment of Au-MSNs and IRE (Figure 2A). The Au-MSNs content was varied from 0 to 100 µg/mL. The membrane permeability was the highest when the combination treatment of 100 µg/mL Au-MSNs and IRE 500 or 800 V/cm was performed, and this observation was correspondent with previous reports [24,25].

Interestingly, penetration of FITC-dextran was dramatically increased under the combined IRE treatment with Au-MSNs. As shown in Figure 2B, penetration of FITC-dextran was increased by 25-fold when the cells were pretreated with Au-MSNs. The penetration of FITC-dextran was greatly affected by the dosing number of Au-MSNs.

### 3.3. Enhanced Cellular Uptake of Au-MSNs under IRE Treatment

In parallel with membrane permeability studies, cellular uptake of Au-MSNs on EMT-6 cancer cells under the IRE treatment was investigated (Figure 2C,D). In this study, cells were co-treated with Au-MSNs and IRE. Data proved that the uptake of FITC-Au-MSNs under IRE treatment was increased by 7-fold when compared with the non-treated control. The result corresponded with cellular membrane permeability studies observed using FITC-dextran.

In this study, we discovered that Au-MSN and IRE treatment displayed a mutual synergistic effect on cell membrane permeability. First, as observed in Figure 2C,D, IRE treatment increased the cellular uptake of Au-MSNs by 7-fold. Following cellular uptake, Au-MSNs then reciprocally increased the membrane permeability in response to IRE treatment. The cellular permeability of EMT-6 cells was increased 25-fold by the Au-MSNs under the IRE treatment, as shown in Figure 2A,B.

### 3.4. Cytotoxic ROS Generation and Lipid Peroxidation under Treatment of IRE and Au-MSNs

ROS are extremely reactive molecules that randomly react with cellular biological molecules, such as proteins, nucleic acids, lipids, and organelles. The antioxidant defense system precisely regulates the cellular content of ROS to maintain an equilibrium state. Excessive ROS production beyond the cellular buffering capacity triggers a cascading pathway of cell death. Consequently, the primary therapeutic function of recently developed adjuvant therapeutic modalities, such as photodynamic therapy, sonodynamic therapy, etc., is to produce excessive cytotoxic ROS in the target tissue.

**Figure 3:**
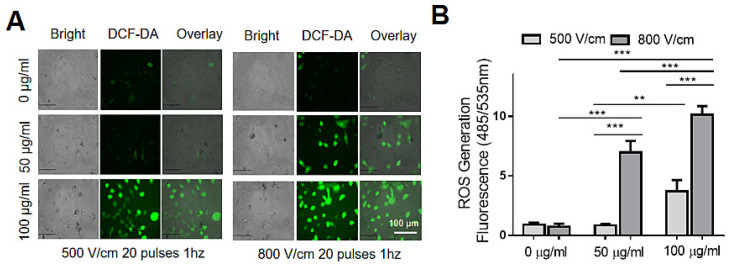
Increased cellular ROS levels under the combination treatment of EMT-6 cells with IRE and Au-MSNs. Cellular ROS was measured using a DCF-DA dye after IRE treatment (500 V/cm or 800 V/cm, 20 plus, 1 Hz) with different amounts of Au-MSNs. (**A**) Fluorescence microscopic images of EMT-6 cells after DCF-DA staining. DCF-DA staining proved cellular ROS was increased by IRE treatment with Au-MSNs. (**B**) Quantification of green fluorescence intensity of EMT-6 cancer cells. Data are provided as means ± standard deviation. Data were tested for significance using two-way ANOVA. Significant differences are indicated by ** p < 0.05, *** p < 0.01 (*n* = 3–5). Increased cellular ROS levels under the combination treatment of EMT-6 cells with IRE and Au-MSNs. Cellular ROS was measured using a DCF-DA dye after IRE treatment (500 V/cm or 800 V/cm, 20 plus, 1 Hz) with different amounts of Au-MSNs. (**A**) Fluorescence microscopic images of EMT-6 cells after DCF-DA staining. DCF-DA staining proved cellular ROS was increased by IRE treatment with Au-MSNs. (**B**) Quantification of green fluorescence intensity of EMT-6 cancer cells. Data are provided as means ± standard deviation. Data were tested for significance using two-way ANOVA. Significant differences are indicated by ** p < 0.05, *** p < 0.01 (*n* = 3–5).

To demonstrate the synergistic effect of IRE and Au-MSNs, we conducted a control cell experiment under specific IRE conditions that did not induce significant ROS generation. Figure 3 shows that single IRE treatment slightly affected the cellular ROS level (Figure 3A,B). However, the combination of Au-MSNs and IRE significantly increased ROS levels, as indicated by the green fluorescence. The combination treatment of 100 µg/mL Au-MSNs and 800 V/cm IRE showed the strongest ROS generation, compared to the treatment of 50 µg/mL Au-MSNs together with 500 V/cm IRE. We hypothesized that during IRE application at 800 V/cm, the ROS production might be elevated via AuNPs in Au-MSNs, which can generate the molecular oxygens by electricity-driven water splitting mediated by Oxygen evolution reactions (OERs) [26].

**Figure 4:**
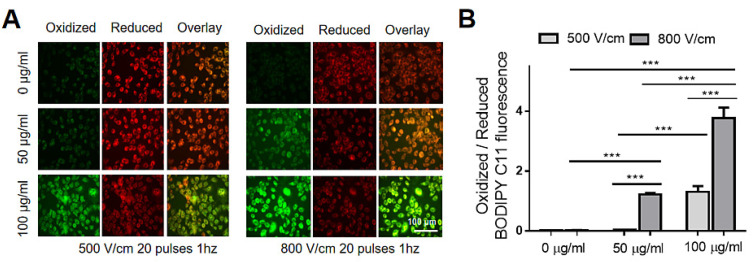
Enhanced cellular lipid peroxidation after IRE treatments (500 V/cm or 800 V/cm, 20 plus, 1 Hz) with different amounts of Au-MSNs. (**A**) Fluorescent microscopic images of EMT-6 cells after IRE treatment with Au-MSNs. Cellular lipid peroxidation was evaluated using a BODIPY™ 581/591 C11 (lipid peroxidation sensor) dye. Microscopic images show that oxidized form of lipid (green) increased by combination treatment. (**B**) Extent of lipid peroxidation. The observed relative fluorescence intensity (green/red) shows that combination treatment of IRE (800 V/cm) and An-MSNs (100 μg/mL) induced highest extent of lipid oxidation. Data are provided as means ± standard deviation. Data were tested for significance using two-way ANOVA. Significant differences are indicated by *** p < 0.01 (*n* = 3–5).

In addition to the ROS generation study, cellular lipid oxidation was assessed using a BODIPY 581/591 C11 dye. Figure 4 shows lipid peroxidation was increased under combination with Au-MSNs in a concentration-dependent manner. Especially, lipid peroxidation was significantly increased after the treatment of 100 µg/mL Au-MSNs and IRE (800 V, 20 pulses, 1 Hz, and 1 ms). These results corresponded with the ROS generation study and indicated that excessive ROS production induced further oxidation of cellular lipids [27,28].

The cell membrane consists of a lipid bilayer and regulates the transport of components for cell growth [13]. Apollonio and his research group suggested that the nanopore formation on the cell membrane is driven by water molecules [10]. Electric pulses push water molecules across the cellular membrane, leading to the creation of transitory membrane holes with inner hydrophobic regions and, ultimately, inducing the reorientation of phospholipids in cell membranes [2,10,28]. Here we assumed that the oxidation of phospholipids in the cell membrane precipitated the formation of irreversible nanopores.

### 3.5. Enhanced Cytotoxic Effect of the Combined Treatment of IRE and Au-MSNs

**Figure 5:**
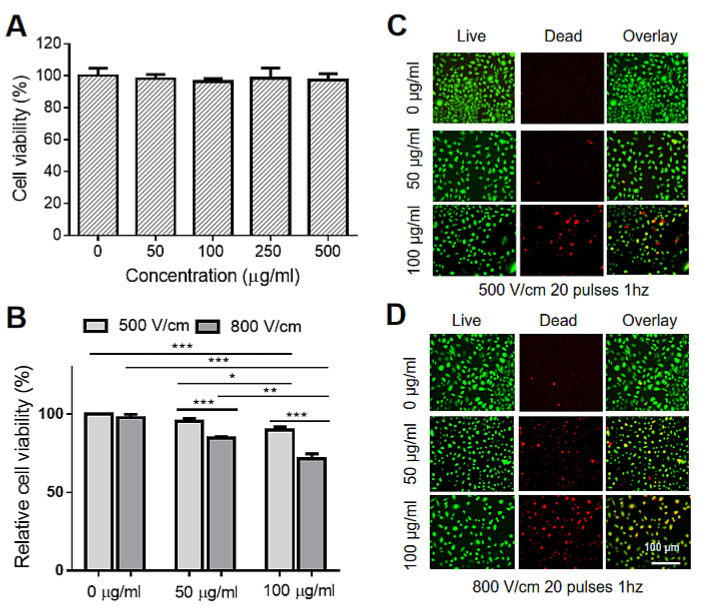
Enhanced cytotoxic effect of IRE on EMT-6 cancer cells under combination treatment with Au-MSNs. (**A**) Innate cytotoxicity of Au-MSNs on EMT-6 cancer cells. (**B**) Relative cell viability of EMT-6 cells after IRE treatment with Au-MSNs. Combined treatment of EMT-6 cells with IRE (800 V/cm) and Au-MSNs (100 μg/mL) showed around 27% of enhanced cytotoxic effect when compared with single IRE (800 V/cm) treatment. (**C**,**D**) Live and dead staining of EMT-6 cells under IRE at 500 V/cm (**C**) and 800 V/cm (**D**) after treating with Au-MSNs. Staining was performed using Calcein-AM (green for live cells) and EthD-1 (red for dead cells) after combined treatment of Au-MSNs and IRE on EMT-6 cells. Data are provided as means ± standard deviation. Data were tested for significance using two-way ANOVA. Significant differences are indicated by * p < 0.1, ** p < 0.05, *** p < 0.01 (n= 3–5).

First, intrinsic cytotoxicity of Au-MSNs on EMT-6 breast cancer cells was observed in this study. As shown in Figure 5A, the CCK-8 assay revealed that Au-MSNs were non-cytotoxic to EMT-6 breast cancer cells at concentrations up to 500 μg/mL. The observed data suggested that Au-MSNs can be used as a non-toxic therapeutic modality in conjunction with IRE.

Then, the therapeutic cytotoxic effects of the combined treatment of Au-MSNs with IRE on EMT-6 cells were investigated. To ensure a combination effect, cells were treated with a combination of non-toxic Au-MSN concentrations (50 and 100 µg/mL) and non-cytotoxic IRE powers (500 and 800 V/cm). As shown in Figure 5B, the cytotoxicity was increased by 27% when a combination treatment of 100 µg/mL Au-MSNs and IRE (800 V/cm) was applied. As compared to IRE 500 V/cm, enhanced cell death was found when 50 and 100 µg/mL of Au-MSNs were treated together with IRE 800 V/cm. Figure 5C proved that the viability of EMT-6 cells was largely affected by the combination treatment of Au-MSNs and IRE. Live and dead staining of EMT-6 cells showed the strongest red fluorescence (Ethd-1, cell death) under the combination treatment of Au-MSNs (100 μg/mL) and IRE (800 V/cm). These results were consistent with previous studies that the combination treatment increased ROS production as well as lipid peroxidation.

## 4. Conclusions

We have demonstrated that IRE, when combined with Au-MSNs, precipitated the generation of ROS for killing breast cancer cells. Combined treatment of IRE with Au-MSNs over the 50 μg/mL concentration more abundantly produced cellular ROS than IRE alone treatment, effectively induced the oxidation of phospholipid of the cell membrane and led to cell death. These combined reactions eventually enhanced the therapeutic functions of IRE treatment. Our findings strongly suggested that Au-MSNs have the potential to improve IRE-mediated tumor ablation.

## Data Availability

The authors confirm that the data supporting the findings of this study are available within the article [and/or] its Supplementary Materials.

## Supplementary Materials

The following supporting information can be downloaded at: https://www.mdpi.com/article/10.3390/bios13010041/s1, Figure S1: Particle size distribution of MSNs; Table S1: Energy dispersive X-ray (EDX) spectroscopy measurement of 12% Au-MSNs; Table S2: Energy dispersive X-ray (EDX) spectroscopy measurement of 5% Au-MSNs.
