# Supplementary material for: Enhanced Therapeutic Potential of Irreversible Electroporation under Combination with Gold-Doped Mesoporous Silica Nanoparticles against EMT-6 Breast Cancer Cells
Source: Biosensors (Basel). 2022 Dec 27;13(1):41. doi: 10.3390/bios13010041 (PMC9855861; doi:10.3390/bios13010041)
Supplement: Supplementary file 1 [file biosensors-13-00041-s001.zip › biosensors-2049086-supplementary.pdf]

biosensors MDPI

Supplementary Information

Enhanced Therapeutic Potential of Irreversible Electroporation under Combination with Gold-Doped Mesoporous Silica Nanoparticles Against EMT-6 Breast Cancer Cells

Yixin Jiang †, Ratchapol Jenjob †, Su-Geun Yang *

Department of Biomedical Science, BK21 FOUR Program in Biomedical Science and Engineering, College of Medicine, Inha University, Incheon 22212, Republic of Korea
* Correspondence: sugeun.yang@inha.ac.kr; Tel.: +82-32-890-2832; Fax: +82-32-890-1199
† These authors contributed equally to this work.

Table S1. Energy dispersive X-ray (EDX) spectroscopy measurement of 12% Au-MSNs.

Element	Wt%	Wt% Sigma	Atomic %
**O**	29.77	0.34	46.39
**Si**	58.76	0.34	52.16
**Au**	11.48	0.26	1.45
**Total**	100.00		100.00

Table S2. Energy dispersive X-ray (EDX) spectroscopy measurement of 5% Au-MSNs.

Element	Wt%	Wt% Sigma	Atomic %
**O**	61.81	0.28	75.43
**Si**	34.88	0.26	24.25
**Au**	3.31	0.17	0.33
**Total**	100.00		100.00

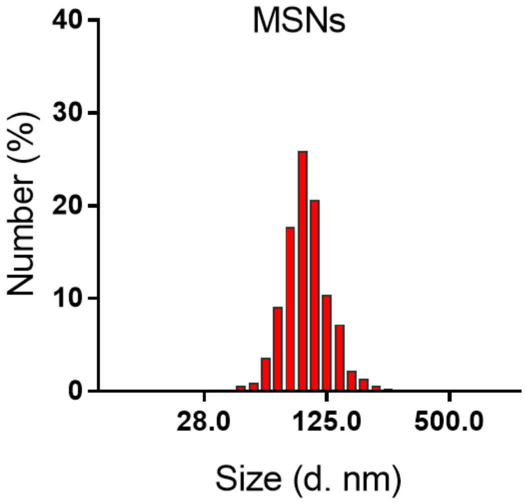

Figure S1. Particle size distribution of MSNs.
